# Acute effects of physical activity breaking up sedentary behavior on cognitive function, biological mechanisms, and practical recommendations: a systematic review

**DOI:** 10.3389/fpsyg.2026.1767939

**Published:** 2026-05-13

**Authors:** Yanqi Li, Yang Jiang, Xinxiao Yin, Yanping Song, Na Yao, Zhen Shen, Qigang Chen

**Affiliations:** 1 School of Physical Education, Yunnan Normal University, Kunming, China; 2 Department of Rehabilitation, Kunming Municipal Hospital of Traditional Chinese Medicine, The Third Affiliated Hospital of Yunnan University of Chinese Medicine, Kunming, China

**Keywords:** brain health, cognitive function, physical activity, sedentary behavior breaks, systematic review

## Abstract

**Introduction:**

Sedentary behavior threatens cognitive health, yet evidence on the effects of physical activity interruptions is inconsistent. This systematic review synthesizes existing findings and clarifies key sources of heterogeneity. It aims to provide evidence-based insights to inform the development of effective interventions that promote brain health.

**Methods:**

Following the PRISMA guidelines, we systematically searched four databases, PubMed, Web of Science, Cochrane, and Scopus, from inception to August 2025 for studies examining the effects of physical activity breaking up sedentary behavior on cognitive function. Three independent researchers performed literature screening, data extraction, and risk-of-bias assessment. A qualitative synthesis was conducted to synthesize the included studies.

**Results:**

A total of 25 studies were included, including 19 randomized crossover trials and 6 randomized controlled trials. These encompassed 31 intervention protocols involving 707 participants. The overall risk of bias across the included studies was moderate. Qualitative synthesis revealed that interrupting sedentary behavior with physical activity acutely improves cognitive function following sedentary periods. Among the 23 studies examining acute effects, 15 (65%) reported significant cognitive improvements following the interruption of sedentary behavior. These improvements were observed most prominently in executive function (reported in 15 of 20 studies) and attention. Among various interventions, interrupting sedentary behavior every 30 min with moderate-to-vigorous intensity walking or stair climbing during prolonged sitting sessions lasting 3–7 h was most effective. However, current evidence is insufficient to establish its chronic impact on cognitive function. Potential mechanisms may primarily involve glycemic control and metabolic regulation; hemodynamic responses and maintenance of cerebral blood flow; and neurotrophic signaling and myokine release following the interruption of sedentary behavior by physical activity. Heterogeneity in the qualitative synthesis was primarily driven by the variations in participant age (ranging from 4 to 77 years), exercise duration (30 s to 1 h), and intervention frequency (ranging from every 4 min to every 3 h).

**Conclusion:**

Interrupting sedentary behavior with physical activity acutely improves cognitive function and promotes brain health. Future studies with longer intervention and follow-up periods are needed to investigate the potential for sustained cognitive benefits.

**Systematic review registration:**

https://www.crd.york.ac.uk/PROSPERO/view/CRD420251162102, Identifier CRD420251162102.

## Graphical abstract

**Figure:**
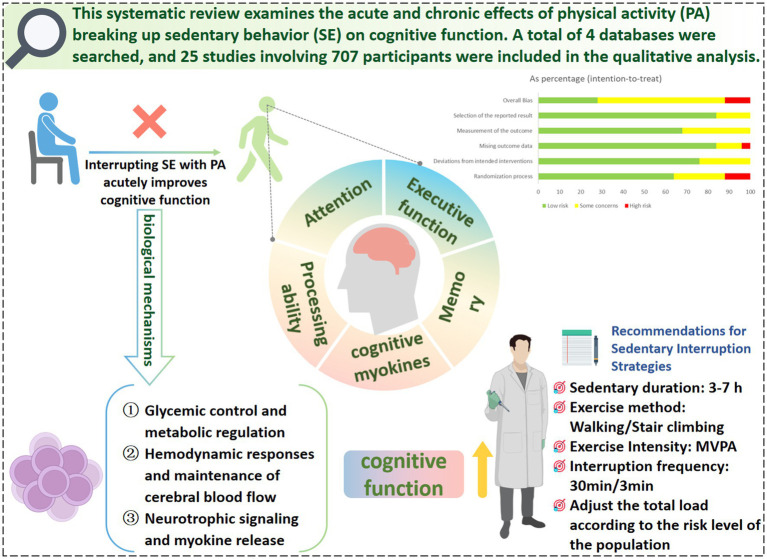

## 1 Introduction

Sedentary behavior (SE) is defined as any activity performed in a static posture, such as sitting, reclining, or lying down, for 30 min or longer while awake, with energy expenditure ≤1.5 metabolic equivalents (METs) (Tremblay et al., 2017; Li et al., 2022). Children and adolescents spend approximately 8 h per day in SE, while adults average 8.8 h per day, with office workers spending 8–12 h per day (Jakubec et al., 2020; Arippa et al., 2023; Chen et al., 2023). SE is associated with adverse health outcomes, including an increased risk of cardiovascular disease, type 2 diabetes, and all-cause mortality (Biswas et al., 2015; Patterson et al., 2018; Ekelund et al., 2019a; Ekelund et al., 2019b). Recent studies have revealed that the disease risks associated with SE have extended to brain health and cognitive function (Raichlen et al., 2023; Zou et al., 2024). Chronic SE is closely linked to dementia risk, with imaging studies showing reductions in brain white matter volume, decreased medial temporal lobe thickness, and mild cognitive impairment (Hamer and Stamatakis, 2014; Arnardottir et al., 2016; Baker et al., 2018; Vancampfort et al., 2018). Therefore, developing scientifically sound and sustainable lifestyle interventions to interrupt SE is particularly crucial for enhancing brain health.

SE interruption is defined as non-sedentary activities that occur between periods of prolonged sitting, breaking up extended sitting sessions, and reducing total sitting time, thereby yielding positive health benefits (Tremblay et al., 2017). Physical activity (PA) is defined as any bodily movement resulting from skeletal muscle contraction that leads to a change in energy expenditure, encompassing exercise or sports behavior (Pieper et al., 2014). It is critical to conceptually distinguish between general PA and interruptions in the SE concept. While traditional exercise interventions focus on accumulating a specific volume of moderate-to-vigorous PA to improve cardiorespiratory fitness, interventions breaking up SE specifically target the detrimental continuity of prolonged sitting. For this review, SE interruption was operationally defined as any intervention involving brief bouts of PA inserted specifically to break up prolonged periods of sitting, rather than general PA accumulation.

As a key modality, SE interruption effectively counteracts the harms of SE and offers numerous well-documented health benefits, making it a crucial strategy for preventing morbidity and mortality (Warburton et al., 2006; Matthews et al., 2015; DEL Pozo-Cruz et al., 2018). Research shows that, under identical conditions, PA breaking up SE improves working memory (WM) in adolescents compared to uninterrupted sitting, while also preventing the decline in prefrontal cerebral blood flow (CBF) that typically follows prolonged sitting (Kjellenberg et al., 2024). However, another study reported that resistance exercise breaks had no significant effect on cognitive function (Charlett et al., 2021). Such discrepancies in findings may arise from differences in key variables, such as the type of PA, the specific cognitive domains assessed, and participant characteristics. Despite initial progress in prior research, significant limitations persist in this field. First, there is considerable heterogeneity in the interruption strategies employed across studies, and there is a lack of systematic exploration of an optimal intervention dose. Second, the underlying biological mechanism remains unclear, as most studies have not adequately explored the links between cognitive improvements and key physiological markers, such as CBF. Third, the majority of current evidence stems from acute studies conducted in specific laboratory settings (Charlett et al., 2021; Kjellenberg et al., 2024), which limits their generalizability to real-world settings and diverse populations.

Due to inconsistencies in participant characteristics, sample sizes, and outcome measures across existing studies, as well as heterogeneity in the control of confounding factors and the choice of statistical methods, a unified research framework to synthesize current evidence remains lacking. Accordingly, this systematic review aims to synthesize evidence on the acute and chronic effects of PA breaking up SE on cognitive function, elucidate sources of heterogeneity across study designs, moderating factors, and outcomes, identify remaining knowledge gaps, and offer evidence-based recommendations for interventions aimed at promoting brain health.

## 2 Materials and methods

### 2.1 Literature search strategy

This study was conducted in accordance with the PRISMA guidelines (Page et al., 2022). The study protocol was prospectively registered with PROSPERO on 7 October 2025 (CRD420251162102). The first author (Yanqi Li) systematically searched four databases, PubMed, Web of Science, Cochrane, and Scopus, covering the period from each database’s inception up to the exact search date of August 1, 2025. The search was restricted to English-language peer-reviewed publications. The search strategy employed Boolean logic, incorporating both free-text terms and controlled vocabulary (e.g., MeSH) to ensure comprehensiveness and accuracy. The exact search strategy applied in PubMed, for example, was as follows: (sedentary behavior OR sedentariness OR sitting OR sedentary lifestyle OR sedentary time OR sedentary OR prolonged sitting OR sitting time) AND (break* OR interrupt* OR fraction* OR intersperse*) AND (physical activity OR exercise* OR training OR activit* OR motion OR aerobic exercise OR resistance training) AND (cogniti* OR cognitive function OR cognitive performance OR memory OR executive function OR reaction times OR accuracy OR attention OR cognitive flexibility OR cognitive inhibition OR information processing speed). In addition, snowball searching (such as citation tracking) was conducted to ensure the literature search was as comprehensive as possible. It is important to note that a preliminary scoping search was conducted in August 2025. This initial search was performed solely to evaluate the feasibility of the review, estimate the volume of available literature, and refine the inclusion and exclusion criteria within the PICOS framework. To maintain the rigorous standards of a systematic review and minimize potential bias, formal data extraction, risk-of-bias assessment, and qualitative synthesis were conducted only after the protocol was finalized and registered in October 2025.

### 2.2 Inclusion and exclusion criteria for literature

Inclusion and exclusion criteria were determined based on the PICOS principle (Higgins et al., 2011).

#### 2.2.1 Inclusion criteria

(1) Population: people of all ages, regardless of gender or health status; (2) Intervention: The intervention must specifically employ PA to break up a controlled period of prolonged sitting (SE interruption). This is operationally defined as inserting at least one short bout of PA into a continuous sedentary period, (SE concepts defined as follows (Edwards and Loprinzi, 2018; Li et al., 2022): sedentary duration >30 min or step count <5,000 steps/day or PA < 150 min/week); (3) Comparison: the intermittent exercise intervention group was compared with the continuous sedentary group, with both groups having equal total duration; (4) Outcome: cognitive function [including at least one measure of cognitive function: attention (ATT), executive function (EF), memory (MEM), processing speed (PS), or cognitive myokines, etc.]; (5) Study design: Randomized controlled trials, randomized crossover studies (with a washout period ≥72 h).

#### 2.2.2 Exclusion criteria

Studies were excluded if they met any of the following criteria: (1) Duplicate publications; (2) Wrong population: literature irrelevant to the target research topic or participants; (3) Wrong intervention: including studies (a) lacking a necessary elution/washout period (for crossover designs), (b) featuring unclear exercise protocols (missing elements of type, intensity, frequency, or duration), or (c) where protocols were altered during the experiment; (4) Wrong outcome: literature that did not report the pre-specified primary or secondary outcomes of interest; (5) Wrong study design or publication type: including review articles, conference abstracts, opinion pieces, brief communications, book reviews, blogs, and other non-peer-reviewed literature; (6) Inaccessible data: literature for which the full text was unavailable.

### 2.3 Literature screening

Duplicate records in the literature were removed using EndNote X9. After the software identified and manually removed duplicate records, the first and second authors (Yanqi Li and Yang Jiang) independently screened and evaluated the literature for eligibility against the inclusion and exclusion criteria. Any disagreements were resolved through discussion or by consulting a third senior reviewer (Xinxiao Yin).

### 2.4 Data extraction and processing

After obtaining full-text articles, the first and third authors (Yanqi Li and Xinxiao Yin) independently extracted data. Extracted content included authors, publication date, study type, participant characteristics (type, sample size, age, and BMI), intervention protocols (mode, duration, frequency, intensity, volume), as well as outcome measures and their results. Study characteristics are presented in tabular format. When encountering data that could not be extracted, attempts were made to contact the original authors, with any discrepancies resolved through consensus reached by the second author (Yang Jiang). Given the substantial heterogeneity observed across studies in participant populations, intervention protocols, and outcome measures, a meta-analysis was deemed inappropriate. Therefore, the findings were synthesized qualitatively.

### 2.5 Risk of bias assessment

Risk of bias assessment will be conducted independently by the first and second authors (Yanqi Li and Yang Jiang) using the Cochrane Risk of Bias 2.0 tool (Rob2) (Higgins et al., 2011). Risk of bias categories include: (1) selection of the reported result; (2) measurement of the outcome; (3) missing outcome data; (4) deviations from intended interventions; (5) randomization process. Any disagreements were resolved through discussion or, if necessary, by consultation with a third author (Xinxiao Yin).

## 3 Results

### 3.1 Search results

**Figure 1:**
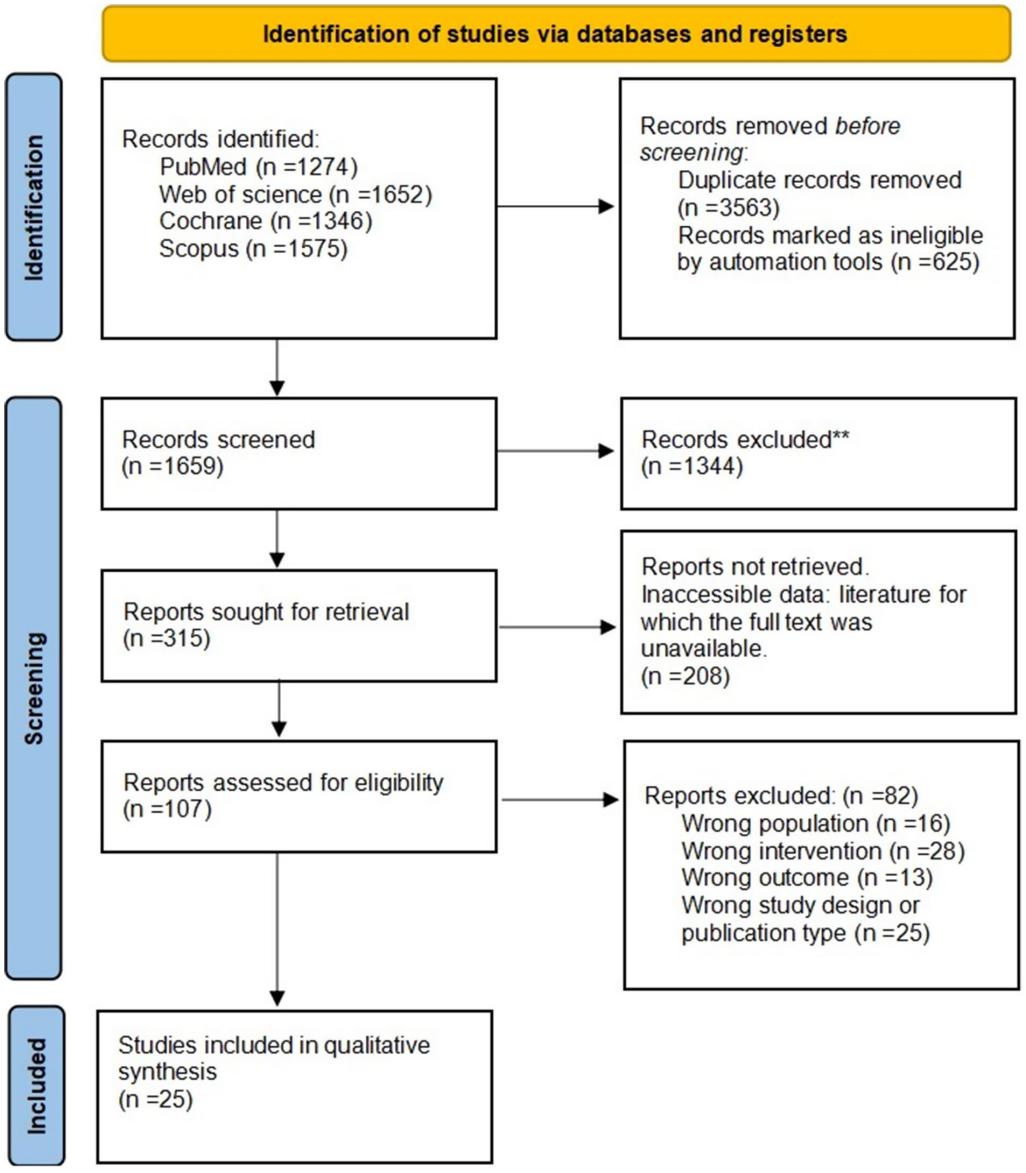
PRISMA flow diagram of the study selection process.

The initial database search identified 5,847 records. After screening, 25 studies met the eligibility criteria and were included in the review. The study selection process is summarized in the PRISMA flow diagram (Figure 1).

### 3.2 Bias assessment results

**Figure 2:**
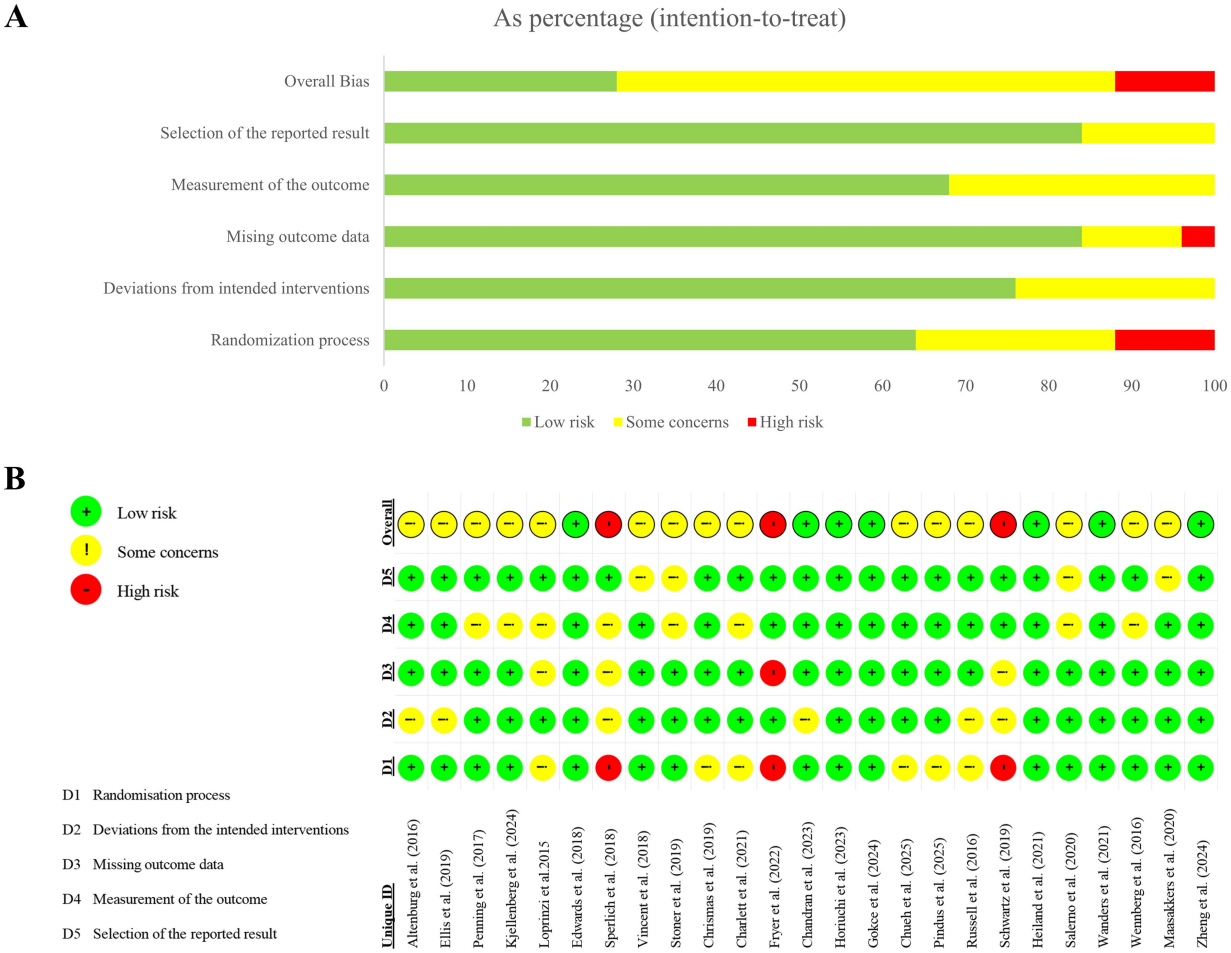
**(A)** Risk of bias summary; **(B)** Risk of bias for the selected RCTs.

The overall risk of bias in the included studies was moderate. A small number of studies were at high risk of bias, while most had potential risk of bias (Figure 2). Most bias domains performed well. However, a few studies did not explicitly describe the randomization procedures used during sequence generation, potentially leading to selection bias. Incomplete reporting of participant numbers or data loss may have contributed to follow-up and reporting biases in a small number of studies.

### 3.3 Basic characteristics of the literature

The characteristics of the included studies are summarized in Table 1, which covers each study’s intervention features, participant profiles, and outcome measures. A total of 25 studies were included, consisting of 19 randomized crossover trials and 6 randomized controlled trials. These 25 studies encompassed 31 distinct intervention protocols, including single-session (*n* = 7) and multiple-session (*n* = 25) interventions. The total sample across all studies was 707 participants, with individual study sample sizes ranging from 6 to 115. Among these, 586 participants were healthy individuals, primarily sedentary populations; 121 were clinical populations, including 70 overweight/obese individuals, 3 underweight individuals, and 48 breast cancer patients. The age range spanned 4–77 years, comprising 206 children and adolescents, 329 adults, and 172 middle-aged and elderly individuals.

**Table 1:** Effects of physical activity and sedentary behavior on cognitive function.

Study		Participants			Intervention protocol					Outcome measures	Main findings
Author (publication year)	Trial type	Type	Quantity	Age (years)/BMI (kg/m^2^)	Method	Duration	Frequency	Intensity	Load		
Children											
1. Altenburg et al. (2016)	RCT	Healthy children	A: 12 males/5 females	11.6 ± 0.9/NR	Dance	4 h	1 session/90 min	MPA: 40-60%HRR	20 min	SELE ATT	For children, interrupting SE with a 20-min MPA improves SELE ATT (B = −0.26, 95% CI [−0.52, −0.004], *p* < 0.05), with two breaks per 90 min being more effective than one (B = −0.29, 95% CI [−0.45, −0.13], *p* < 0.05).
			B: 11 males/9 females	11.4 ± 0.8/NR			2 sessions/90 min				
			Control group: 7 males/12 females	11.7 ± 0.7/NR			None				
2. Ellis et al. (2019)	RCT	Healthy children	Intervention group: 47 males/8 females	4.1 ± 0.7/16.4 ± 1.4	Standing/Walking/Stretching	12 weeks	>180 min/school day	LPA	1-2 min	EF, INH, WM, FLEX	Implementing 3 h of standing-desk LPA daily to interrupt children’s SE is feasible and acceptable, but it has no significant effect on the EF of sedentary children (*p* > 0.05).
			Control group: 40 males/20 females	4.2 ± 0.6/16.3 ± 1.3							
Adolescents											
3. Penning et al. (2017)	Cross RCT	Healthy 11/Overweight 7	males/7 females	13.5 ± 0.9/21.1 ± 3.9	Standing/Stretching	6 h	20 min	LPA	2–4 min	ATT	Reducing SE time by 50% through intermittent activity sessions of 2–4 min every 20 min significantly improves cardiovascular health and metabolism in adolescents, while enhancing cognitive function and ATT (*p* = 0.15; *d* = 0.54).
4. Kjellenberg et al. (2024)	Cross RCT	Healthy 13/Overweight 1/Underweight 3	6 males/11 females	13.6 ± 0.7/18.9 ± 2.1	A: Simple resistance activities 1	80 min	17 min	MPA RPE: 9 ± 2	3 min	WM	Short bursts of MPA interspersed with SE can improve WM in adolescents. Engaging in 3 min of resistance exercise every 17 min (*p* = 0.01) is more effective than performing stepping exercises (*p* = 0.02).
					B: Stepping			110 times/min			
Adults											
5. Loprinzi and Kane (2015)	Cross RCT	Healthy adults	51 males/36 females	21.4 ± 2.1/23.3–24.9	Treadmill walking	30 min	Single session	Low: 40–50%HRmax	30 min	ATT, FLEX, MEM, REA	SE is negatively correlated with ATT. Interrupting SE with a single 30-min MPA session acutely enhances ATT in cognitive function (*p* = 0.004).
								Moderate: 51-70%HRmax			
								vigorous: 61-85%HRmax			
6. Edwards and Loprinzi (2018)	RCT	Healthy adults	Intervention group: 8 males/15 females	21.74 ± 2.82/25.14 ± 6.77	Normal PA	After 1 week of SE	>150 min/week	MVPA	>150 min/week	WM, ATT, REA	After 1 week of SE intervention, No significant decline was observed on cognitive function tests, suggesting that short-term SE may not impair cognition (*p* > 0.05).
			Control group: 4 males/6 females	22 ± 2.79/26.16 ± 4.06							
7. Sperlich et al. (2018)	Cross RCT	Healthy adults	5 males/7 females	22 ± 2/21.7 ± 2.1	HIIT: Callisthenic exercises	3 h	Once after 60 min	VPA: As Much as Possible	6 min	EF, ATT, INF	A single 6-min bout of HIIT, used to interrupt SE, had no significant effect on cognitive function (*p* > 0.05).
8. Vincent et al. (2018)	Cross RCT	Healthy adult males	6 males	27 ± 3.7/NR	Walking	7 h	30 min	3.2 km/h	3 min	RES SPE, ALE	Taking 3-min walking breaks every 30 min to interrupt SE improves subjective sleepiness and enhances ALE (*p* < 0.001) in adult males after 3 days of sleep restriction (5 h per night), but does not affect other cognitive functions (*p* > 0.05).
9. Stoner et al. (2019)	Cross RCT	Healthy adult males	6 males/14 females	21.7 ± 2.5/25.5 ± 6.1	Calf raises	3 h	10 min	20 times/min	30s	EF	SE for 3 h did not significantly affect EF. Interrupting SE with 10-min intervals of intermittent calf raises did not improve EF (*p* = 0.83).
10. Chrismas et al. (2019)	Cross RCT	Healthy adult women	11 females	21–44/NR	Treadmill walking	7 h	30 min	MVPA: RPE 12–14, 5.0–8.3 km/h	3 min	EF, RT, CRT, ATT	Interrupting SE with 3 min of moderate-intensity walking every 30 min significantly improves ATT and EF in Qatari women (p = 0.01).
11. Charlett et al. (2021)	Cross RCT	Healthy adults	5 males/7 females	25 ± 6/24.7 ± 4.9	Combined resistance exercise	5 h	30 min	LPA, Comfortable Pace, RPE: 7.9 ± 1.4	3 min	ALE, RT, PM	Breaking up SE with 3 min of resistance exercise every 30 min does not improve cognitive function (*p* ≥ 0.05).
12. Fryer et al. (2022)	Cross RCT	Healthy adult males	13 males	21.4 ± 1.7/23.9 ± 2.5	Leg shaking	3 h	4 min	250 times/min	1 min	EF	Interrupting SE with 1 min of leg shaking every 4 min can improve EF decline in adults caused by SE after consuming Western-style high-fat, high-sugar meals (MD = 2.3 s, *d* = 0.97).
13. Chandran et al. (2023)	Cross RCT	Healthy adults	17 males/4 females	26.65 ± 2.64/22.32 ± 2.46	A: Walking	4 h	1 h	LPA: RPE 11	3 min	EF, RT, ACC	Breaking up SE with 3 min of light to moderate PA every hour can improve cognitive RT (*p* < 0.001) and ACC (*p* < 0.005) associated with SE, as well as enhance EF.
					B: Stair Climbing			MPA: RPE 13			
14. Horiuchi et al. (2023)	Cross RCT	Healthy Adults	11 males/9 females	21 ± 1/21.6 ± 1.6	Half squat + calf raise	3 h	20 min	15 times/min	1 min	EF, RT	Performing 1 min of half-squats every 20 min to break up SE can mitigate EF decline in adults caused by SE (*p* = 0.048), maintain CBF, and reduce mental fatigue and ATT deficits.
15. Gökçe et al. (2024)	Cross RCT	Healthy adults	9 males/14 females	22.78 ± 2.87/23.31 ± 3.37	HITT: Stationary bicycle	23 min	Single session	50–80 revolutions/min	23 min	WM, RT, ACC, BDNF, CTSB	Single-session bicycle HIIT improves WM (*p* = 0.016) in young adults and increases serum BDNF (*p* = 0.013) and CTSB (*p* = 0.019) levels, but has no significant effect on motor cortex excitability.
16. Chueh et al. (2025)	Cross RCT	Healthy adults	18 males	25 ± 4/23.5 ± 3.2	Walking	3.5 h	30 min	6.4 km/1 h	3 min	RT, ACC, WM, P3 amplitude	Taking a 3-min walk every 30 min to break up SE can increase P3 amplitude (*p* = 0.041), improve ATT, and lower postprandial blood glucose levels (*p* = 0.028).
17. Pindus et al. (2025)	Cross RCT	Obese adults	7 males/12 females	29.9 ± 7.5/30.0 ± 3.64	Walking	3 h	30 min	55%HRR	3.5 min	RT, ACC, WM, P3b amplitude	Interrupting SE with 3.5 min of moderate-intensity walking every 30 min reduces P3b amplitude latency (*p* = 0.037) and improves ATT, but does not improve WM (*p* ≥ 0.23).
Middle-aged person											
18. Russell et al. (2016)	RCT	Healthy Middle-aged adults	10 males/26 females	40.08 ± 11.93/NR	Standing work	5 days	1 h/day	LPA	1 h	CRT, ATT, WM, IPS	Using a standing desk for 1 h per workday to interrupt SE had no significant effect on ATT, IPS, WM, or task efficiency (all d < 0.2).
19. Schwartz et al. (2019)	RCT	Healthy adults-middle-aged individuals	10 males/8 females	36.3 ± 10.3/23.1 ± 1.8	Standing work	23 weeks	Per workday	LPA	Every workday	WS, ATT, RT	Using sit-stand work to interrupt SE had no significant effect on cognitive performance (all *p* > 0.05).
20. Heiland et al. (2021)	RCT	Healthy middle-aged adults	8 males/5 females	50.5 ± 4.6/24 ± 2.4	A: Walking	3 h	30 min	HRmax: 70–80%	3 min	RT, ALE	Interrupting SE with 3 min of moderate-intensity walking every 30 min, rather than resistance exercise, can improve RT and ALE impaired by SE (*p* ≤ 0.05)
					B: Simple Resistance activities						
21. Salerno et al. (2020)	Cross RCT	Middle-aged breast cancer patients	48 females	56.02 ± 10.99/NR	Walking	A:10 min	一次	HRmax: 60% RPE: 8–12	A:10 min	EF, INH, WM, FLEX	Interrupting SE with moderate-intensity walking every 10/20/30 min improves EF in SE breast cancer patients (*p* > 0.05), with 20-min walks yielding the greatest cognitive benefits (*d* = −0.24).
						B:20 min			B:20 min		
						C:30 min			C:30 min		
Elderly											
22. Wanders et al. (2021)	Cross RCT	Middle-aged and elderly obese individuals	5 males/19 females	59.6 ± 8.1/30.2 ± 2.5	Bicycle	4 h	30 min	HRmax: 50–70%	5 min	ATT, EF, RT, WM	Interrupting SE with 5 min of moderate-intensity cycling every 30 min does not improve cognitive function in obese individuals during SE (all *p* > 0.05).
23. Wennberg et al. (2016)	Cross RCT	Middle-aged and elderly obese individuals	10 males/9 females	45–75/31.5 ± 4.7	Electric treadmill	7 h	30 min	LPA: 3.2 km/h, RPE: 9.1 ± 2.0	5 min	EM, EF	Interrupting SE with 5 min of moderate-intensity walking every 30 min does not improve EF (*p* > 0.05) and ME (*p* = 0.077) in obese individuals.
24. Maasakkers et al. (2020)	Cross RCT	Healthy elderly	13 males/9 females	78 ± 5.3/26 ± 4.0	Walking	3 h	30 min	LPA: Normal walking speed	2 min	EF, WM	Interrupting SE with 2 min of walking every half hour did not affect cognitive function in older adults (all *p* > 0.05).
25. Zheng et al. (2024)	Cross RCT	Healthy elderly	9 males/17 females	67.8 ± 11.3/27.8 ± 5.4	Walking	5d, 30 min/d	Once	MPA: 100 steps/min, 1.12 m/s	30 min	ATT, INH, EF, IPS	30 min of walking daily can reduce SE in physically inactive older adults, thereby improving EF (p ≤ 0.05) and ATT (p ≤ 0.05).

Min, minutes; h, hours; d, days; km, kilometers; m, meters; HIIT, high-intensity interval training; PA, physical activity; LPA, light physical activity; MPA, moderate physical activity; VPA, vigorous physical activity; MVPA, moderate-to-vigorous physical activity; RPE, rate of perceived exertion; HRR, heart rate reserve; HRmax, maximum heart rate; Simple resistance activities1, three sets of half-squats, calf raises, and gluteal contractions; Callisthenic exercises, squats, skipping, and jumping jacks; Combined resistance exercise, half-squats, upright wall push-ups, knee raises and calf raises; Simple Resistance activities2, three rounds of seven half-squats, nine calf raises, six alternating knee raises with gluteal contractions after each knee raise; ATT, attention; SELE ATT, select Attention; ALE, alertness; P3/P3b amplitude, attentional neuroelectric outcomes; EF, executive function; INH, inhibition; FLEX, flexibility; WM, working memory; CRT, choice reflection time; RT, reflection time; MEM, memory; EM, episodic memory; PM, probe memory; ACC, accuracy; PS, processing speed; REA, reasoning; RES SPE, reaction speed; IPS, information processing speed; WS, working speed; BDNF, brain-Derived Neurotrophic Factor; CTSB, cathepsin B; CBF, cerebral blood flow; B, beta coefficient; CI, confidence interval; *p*, probability value; *d*, Cohen’s *d*; MD, mean difference.

Included cognitive function indicators comprise: (1) Attention (ATT): Select Attention (SELE ATT), Alertness (ALE), Attentional neuroelectric outcomes (P3/P3b amplitude); (2) Executive function (EF): Inhibition (INH), Flexibility (FLEX), Working memory (WM), Choice Reflection Time (CRT), Reflection Time (RT); (3) Memory (MEM): Episodic memory (EM), probe memory (PM), Accuracy (ACC); (4) Processing speed (PS): Reasoning (REA), Reaction speed (RES SPE), Information processing speed (IPS), Working speed (WS); (5) Cognitive myokines (myokines related to exercise-induced cognitive improvement): Brain-Derived Neurotrophic Factor (BDNF), Cathepsin B (CTSB).

Twenty three of the included studies investigated the acute effects on cognitive function following interruptions of SE by PA. The interventions included dancing, standing, walking, stretching, bodyweight resistance training, stepping, aerobics, calf raises, leg shakes, stair climbing, half-squats, and cycling. Exercise duration ranged from 30 s to 1 h, with intensity levels categorized as low, moderate, or vigorous. The frequency of interruptions varied from once every 4 min to once every 3 h. Fifteen of these studies reported acute cognitive improvements following the interruption of SE by PA. The specific intervention protocols associated with positive outcomes are detailed in Table 2.

**Table 2:** Specific effective intervention programs for acutely improving cognitive function through PA breaking up SE.

Primary variables	Specific form	References Table 1
Participants	Healthy individuals: children, adolescents, adults, middle-aged, and elderly individuals	1, 3, 4, 5, 8, 10, 12, 13, 14, 15, 16, 20, 25
	Clinical population: obese, underweight, and breast cancer patients	3, 4, 5, 21
Exercise methods	Walking	5, 8, 10, 13, 16, 17, 20, 21, 25
	Simple resistance activities1, Stepping	4
	Dance; Standing/Stretching	1; 3
	Legs shaking; Climbing stairs	12; 13
	Half squat + Calf raises; HIIT cycling	14; 15
Exercise intensity	LPA	3, 8, 12, 13
	MPA-MVPA	1, 4, 5, 10, 13, 14, 15, 16, 17, 20, 21, 25
Duration of SE	3–7 h	1, 3, 8, 10, 12, 13, 14, 15, 16, 17, 20, 25
	10–80 min	4, 5, 21
Intermittent frequency	3–3.5 min/30 min	8, 10, 16, 17, 20
	1–4 min/20 min	3, 14
	3 min/17 min; 1 min/4 min; 3 min/1 h	4; 12; 13
	Once before and once after 90 min	1
	Single session: 1 session, 20–30 min	5, 15, 21, 25

EF was the most extensively studied cognitive domain, examined in 20 of the included studies. The majority of these studies (*n* = 15) reported that PA breaking up SE led to improvements in one or more subdomains of EF (Chrismas et al., 2019; Salerno et al., 2020; Heiland et al., 2021; Fryer et al., 2022; Chandran et al., 2023; Horiuchi et al., 2023; Gökçe et al., 2024; Kjellenberg et al., 2024; Zheng et al., 2024). In healthy populations, the greatest improvement in EF was observed after interrupting 7 h of SE with 3-min bouts of moderate-intensity walking every 30 min, accompanied by significant changes in CRT and RT (Chrismas et al., 2019). In clinical populations, interrupting SE every 20 min with a single bout of moderate-intensity walking, compared to 20 min of continuous sitting, resulted in the greatest improvement in EF (particularly INH, WM, and FLE) (Salerno et al., 2020). ATT is the cognitive indicator receiving the second-highest level of attention in this research field, after EF, with 15 studies addressing it. Most studies reported that PA breaking up SE produced positive improvements in one or more ATT metrics (Loprinzi and Kane, 2015; Altenburg et al., 2016; Penning et al., 2017; Vincent et al., 2018; Chrismas et al., 2019; Heiland et al., 2021; Zheng et al., 2024; Chueh et al., 2025; Pindus et al., 2025). For ATT, the greatest improvement was observed following a 3- or 3.5-min bout of moderate-intensity walking every 30 min during 3- or 3.5-h sedentary periods, and both intervention schedules also showed significant changes in P3/P3b amplitude (Chueh et al., 2025; Pindus et al., 2025). Positive effects on MEM, cognitive myokines, and PS were also observed in a limited number of studies (Chandran et al., 2023; Gökçe et al., 2024; Zheng et al., 2024).

In contrast, only two studies investigated the chronic effects of PA breaking up SE on cognitive function. The interventions involved standing, stretching, and walking; the duration ranged from 180 min to one workday; the intensity was LPA; and the frequency was every workday or school day. Results showed no improvement in ATT, EF, MEM, or PS (Ellis et al., 2019; Schwartz et al., 2019).

## 4 Discussion

This systematic review shows that PA breaking up SE can acutely enhance cognitive function. Positive acute effects were most consistently observed for EF and ATT; there was some evidence of benefits for MEM, PS, and cognitive myokines. But evidence regarding the efficacy of long-term interventions for sustained cognitive enhancement remains limited.

### 4.1 Summary of evidence

One early study indicated that while interrupting SE improves performance on specific cognitive tasks compared to continuous sitting, it has no significant effect on overall cognitive function (Li et al., 2022). Following this, Feter et al. (2024) extended the findings, showing that PA breaking up SE, in either a single or multiple bouts, acutely enhances cognitive performance measured 3 h to 3 d following the interruption, and that multiple interruptions are more effective than a single interruption. However, evidence regarding the chronic effects of these interruptions is inconclusive. To summarize the results, I reviewed and aligned them with previous research and support the conclusion that PA provides acute cognitive benefits for interrupting SE. Although our findings on acute cognitive benefits are consistent with prior reviews, we hope this work offers additional insights by exploring areas that have not yet been fully addressed. Previously, evidence on the effects of PA interruptions remained inconsistent, with some trials reporting no cognitive benefits. Our review clarifies these discrepancies by systematically exploring the sources of heterogeneity, specifically identifying how parameters such as intervention modality, intensity, and frequency moderate cognitive outcomes. Furthermore, unlike previous reviews that primarily focused on behavioral outcomes, this study provides a comprehensive synthesis of the underlying biological mechanisms. It translates these findings into specific, evidence-based practical recommendations for different populations.

Though the extant literature tends to support the beneficial impact of PA on interrupting SE, those that report non-significant outcomes also provide equally valuable insights. First, the specific parameters of the interruption protocols are key moderating variables influencing their efficacy. In terms of PA intensity, MPA provides the strongest support for improving cognition. For example, Loprinzi and Kane (2015) examined the effects of low-, moderate-, and vigorous-intensity walking intervals. They found that just 30 min of moderate-intensity walking improved ATT test scores. Similar to the above, cognitive improvements were observed during 3 h of prolonged sitting with intermittent walking with MPA (Pindus et al., 2025) but not with LPA (Maasakkers et al., 2020). As for the activity mode, whole-body aerobic exercise seems better than local resistance training at improving cognition. Heiland et al. (2021) found no cognitive benefits of localized resistance exercise compared with walking on the same interruption schedule; in contrast, a study showed that both walking and climbing stairs enhance cognitive functions (Chandran et al., 2023). Therefore, future studies may investigate the use of an integrated whole-body resistance training program (both upper and lower limbs) to interrupt SE, focusing on cognitive benefits and determining the optimal dose across activity modalities. Regarding interruption frequency, the vast majority of included studies show that higher frequencies are better, e.g., 1–3.5 min of activity every 20–30 min. But there are positive results too, when the interruptions are at a lower frequency (like every 60 min, 3 min each time), and even for just one longer bout (like 20–30 min). Based on the observed trends across the included studies, we tentatively infer that a nonlinear, potentially inverted U-shaped dose–response relationship may exist between the frequency (or duration) of SE interruption and cognitive benefits. While the current qualitative synthesis does not permit a formal regression analysis to confirm this non-linear trend statistically, this inference aligns with the Yerkes-Dodson principle (Beerendonk et al., 2024), suggesting that either insufficient or excessive stimulation may be suboptimal for cognitive arousal. Future research employing a standardized design, meta-regression, or dose–response meta-analysis is warranted to validate this hypothesis empirically.

Second, the varied results from the included studies underscore the need to fine-tune our understanding of the SE-cognition association. This relationship seems to depend on context, since not every type of SE is equally harmful to the brain. Recent studies suggest that SE does not have inherent absolute benefits or harms for the brain; it really depends on the specific context and task (Zhang et al., 2025). Mentally active SE often has a positive relationship with brain health, such as good reading (Cristi-Montero et al., 2023; Wingood et al., 2024), but mentally passive SE may result in negative outcomes and an increased risk of diseases such as dementia, such as aimless TV watching (Raichlen et al., 2022; Wingood et al., 2024). In support, an 8,125 adolescent cross-sectional study found that time spent on sedentary reading was positively associated with better cognitive performance and larger cortical surface area in higher-order cognitive areas (frontal/temporal lobes); on the other hand, television watching time correlated with worse cognitive function and thinning of the cortex, in places like the lateral temporal lobe (Rauschecker et al., 2025). Neuroimaging evidence also supports the view that even screen-based SE can be beneficial for youth when they are cognitively engaged (e.g., problem-solving games), a state associated with positive brain structure and function (Zhang et al., 2025). Therefore, future research and public health interventions should shift their focus from merely reducing sedentary time to improving the quality of sedentary time by encouraging mentally active SE. Moreover, people who mainly do mentally passive SE should be regarded as a high-risk group to focus on.

### 4.2 Biological mechanisms

**Figure 3:**
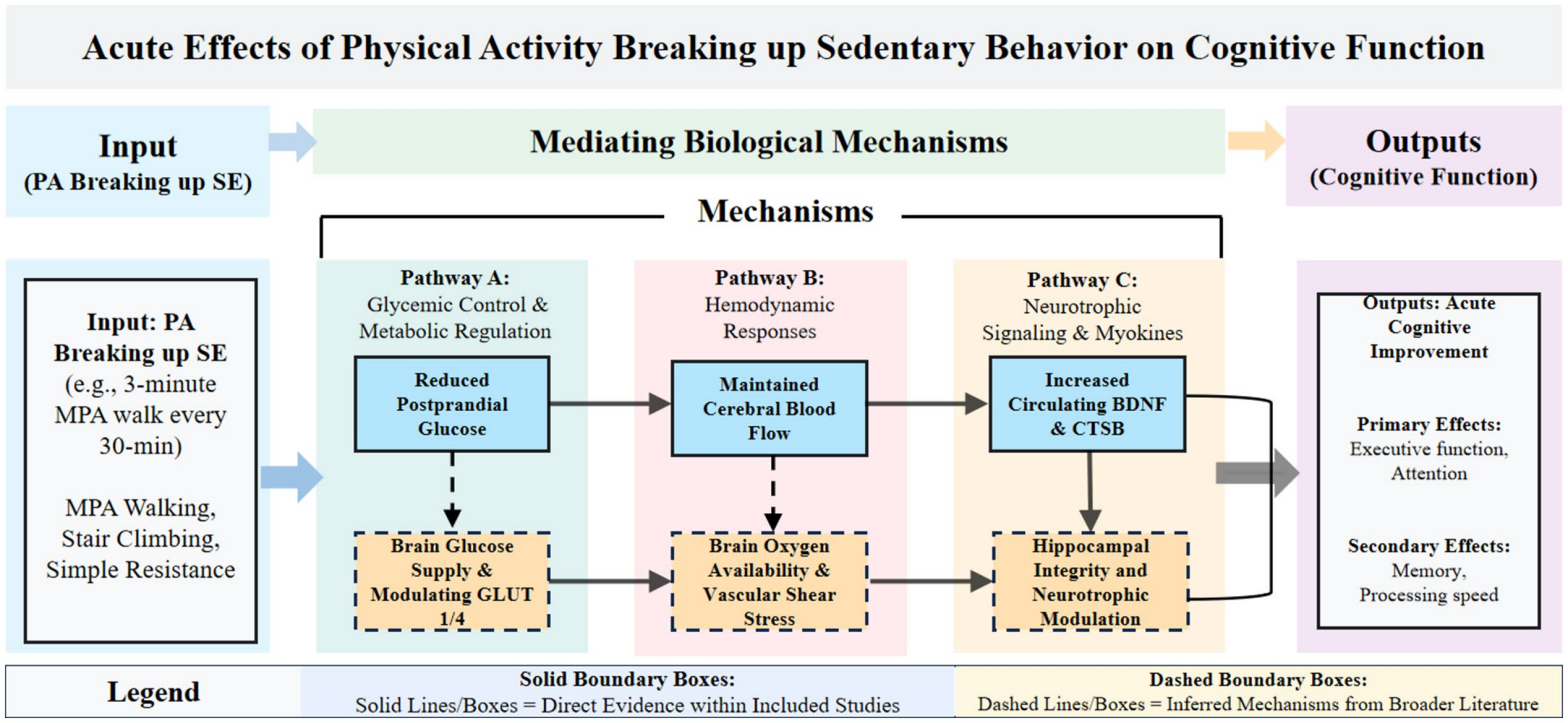
Biological mechanisms.

The positive cognitive outcomes observed in this review should be interpreted in the specific context of interrupting SE rather than in the context of general exercise training. Among the empirical studies included in this review, several directly reported improvements in physiological and biochemical markers following the interruption of SE. Breaking up SE has been shown to maintain CBF (Horiuchi et al., 2023), significantly reduce postprandial blood glucose levels (Chueh et al., 2025), and notably increase serum levels of BDNF and CTSB following brief interventions (Gökçe et al., 2024). Although current empirical research on SE interruption primarily captures improvements such as peripheral glycemic control, maintenance of blood flow, and elevated serum neurotrophic factors, the broader exercise physiology literature suggests that these observed benefits are linked to deeper neurobiological mechanisms. Specifically, the ultimate cognitive enhancements are likely further mediated by the increased transport efficiency of glucose transporter 1 (GLUT1) at the blood–brain barrier, optimized central hemodynamics, and enhanced synaptic plasticity in the hippocampus. The biological mechanisms by which PA improves cognitive function in SE constitute a complex, multidimensional process, primarily involving three aspects: glycemic control and metabolic regulation; hemodynamic responses and maintenance of cerebral blood flow; and neurotrophic signaling and myokine release (Figure 3).

#### 4.2.1 Glycemic control and metabolic regulation

Glucose mainly crosses the blood–brain barrier via glucose transporter 1 (GLUT1) and moves into brain tissue down its concentration gradient to be used by neurons (Patching, 2017). However, SE causes postprandial hyperglycemia, hyperinsulinemia, and insulin resistance, thereby disrupting glucose and lipid metabolism (Blankenship et al., 2014; Dempsey et al., 2018). It impairs blood–brain barrier function, altering permeability and reducing GLUT1 transport efficiency, leading to functional hypoglycemia in the brain with inadequate energy supply (Wheeler et al., 2017). In people who have subclinical diabetes, high blood sugar (hyperglycemia), and problems with the way their body uses insulin (insulin resistance) have also been connected to a drop in thinking skills and shrinking of the part of the brain that helps with MEM (Weinstein et al., 2015; Xu et al., 2015). PA breaking up SE can regulate transporters, thereby interrupting the vicious cycle. Peripherally, activity increases glucose uptake into skeletal muscle through contraction, as well as through insulin. It is mainly achieved by ensuring that GLUT4 translocates from intracellular compartments to the cell membrane, thereby increasing the amount of glucose that enters the muscles, promoting glycogen synthesis, and helping to maintain steady blood sugar levels throughout the body (Roeckl et al., 2008). Central to increasing GLUT1 function at the blood–brain barrier and to help normalize the normal physiological gradient of glucose concentration, thus allowing efficient delivery of glucose to the brain and the provision of an adequate energy source to sustain and possibly enhance cognitive function (Chandrasekaran et al., 2021).

#### 4.2.2 Hemodynamic responses and maintenance of cerebral blood flow

Sustained brain function requires a steady supply of oxygen and glucose to the brain via CBF. Prolonged sitting with a flexed posture at the hip and knee joints leads to venous pooling in the lower limbs, higher blood viscosity, and slower arterial blood flow (Gruhn et al., 2001). This decreases venous return and cardiac output, thereby lowering cerebral perfusion (McManus et al., 2015). At the same time, the reduction in lower-limb vascular shear stress caused by muscle inactivity leads to dysfunction of endothelial cells (Kruse et al., 2018). Disruption of peripheral vasculature homeostasis results in adverse effects on central hemodynamics and cerebral cortical circulation, worsening of cerebral hypoperfusion (Hachiya et al., 2010). Chronic cerebral hypoperfusion decreases oxygen supply to the brain, disrupts neuronal metabolism, damages neuronal and glial cell function, and causes cognitive deficits, including WM impairment and learning deficits (Watts et al., 2018). Critical brain regions for MEM and EF, like the temporal and frontal lobes, are highly susceptible to such hypoperfusion (Carter et al., 2018; Carter et al., 2019; Chandrasekaran et al., 2021; Zheng et al., 2024). PA breaking up SE counteracts these effects by disrupting the static postural pattern. The resulting muscle activation decreases neuromuscular inhibition, improves venous return, and increases cardiac output, thereby maintaining CBF (Mailey et al., 2017; Carter et al., 2018). More importantly, muscle contraction provides a mechanical stimulus that acutely raises vascular shear stress. Stimulating the endothelial cells to release vasodilatory factors that cause the blood vessels to relax and improve blood flow (Faraci and Heistad, 1998). Research indicates that intermittent time spent standing substantially improves the ability of blood flow to dilate vessels compared with SE (Paterson et al., 2020). And this improved peripheral vascular performance works together with the heart to increase cardiac output, which in turn protects CBF and provides a hemodynamic base sufficient for normal brain metabolism (McManus et al., 2015).

#### 4.2.3 Neurotrophic signaling and myokine release

SE impairs the integrity of brain structure and the neurochemical environment (Siddarth et al., 2018). Hippocampus is an important structure in the medial temporal lobe, playing a significant role in learning and MEM, which is highly atrophic in neurodegenerative diseases like AD (Raz et al., 2005; Cotman and Berchtold, 2007). Research suggests SE is negatively correlated with hippocampal thickness, which may be due to decreased CBF, reduced synaptic plasticity, and reduced neurotrophic support from inactivity (Jiang et al., 2016; Siddarth et al., 2018). In addition, BDNF is a key protein mainly secreted by the hippocampus, plays a vital role in neuronal survival and synaptic plasticity, and is essential for the brain to carry out cognitive functions (Barde, 1994). SE may not only affect hippocampal function and reduce BDNF secretion, but also worsen fatigue and cognitive impairment through inflammation and oxidative stress (Massaad and Klann, 2011). BDNF and CTSB are two muscle factors associated with cognitive enhancement that are very strongly correlated with exercise (Pedersen, 2019). Research shows that acute physical exercise can elevate BDNF when we are cognitively better than normal (Hwang et al., 2016). Exercise directly stimulates the hippocampus to produce more BDNF (Vaynman et al., 2004). At the same time, muscle contractions release messenger molecules called “myokines” (such as CTSB and irisin) that are transported in the bloodstream to the brain and indirectly spur BDNF (Wheeler et al., 2020). Also, PA increases neurotransmitter levels, including dopamine and catecholamines. Improving ALE and arousal levels creates a good neurochemical environment for improving cognition (McMorris, 2016).

### 4.3 Preliminary practical implications

Once we figure out exactly how PA affects SE to improve cognition, creating specialized exercise plans to break up SE will become very important. Based on the synthesis of the 25 empirical studies included in this review, we offer the following preliminary implications for breaking up SE to enhance acute cognitive function (Table 3). It is important to note that these insights are primarily derived from acute intervention trials, and their long-term efficacy remains to be fully established. And it is crucial to recognize that the optimal dose for cognitive benefits is population-specific.

**Table 3:** Factors influencing cognitive outcomes of sedentary interruption interventions and recommended approaches.

Main factors	Key variables	Recommendation	References Table 1
Interrupted protocol	Exercise methods	Walking/Stair climbing	5, 8, 10, 13, 16, 17, 20, 21, 25
	Exercise intensity	MPA-MVPA	1, 4, 5, 8, 11, 13, 15, 16, 17, 20, 21, 25
	Duration of SE	3–7 h	8, 10, 12, 13, 14, 16, 17, 20, 25
	Intermittent frequency	3 min/30 min	8, 10, 16, 17, 20

For healthy young and middle-aged populations, the current evidence suggests that frequent, short-duration PA interruptions are an effective strategy for immediate cognitive enhancement. Specifically, performing approximately 3 min of moderate-intensity walking every 30 min appears to be a robust protocol for improving cognitive function (Vincent et al., 2018; Chrismas et al., 2019; Heiland et al., 2021; Chueh et al., 2025; Pindus et al., 2025).

Evidence regarding clinical populations, such as individuals with obesity or breast cancer survivors, suggests that the dose of interruption may need to be adjusted to account for different physiological baselines. For instance, a higher frequency of interruptions (e.g., every 20 min) might be necessary to elicit cognitive benefits in breast cancer survivors (Salerno et al., 2020). Similarly, for individuals with metabolic impairments, the focus should remain on interruptions that prioritize glycemic control, as postprandial glucose regulation is a key mediator of cognitive performance in this group (Pindus et al., 2025). However, due to the limited number of studies in these cohorts, these strategies should be applied with caution.

To translate these findings into actionable guidelines for clinicians and occupational healthcare workers, we recommend integrating “SE interruption” into standard behavioral prescriptions. For healthy adults or office workers, clinicians should prescribe brief, 3-min bouts of moderate-intensity walking every 30 min of sitting as a baseline preventative strategy for maintaining cognitive arousal and brain health. For clinical or rehab populations (e.g., metabolic or breast cancer patients), clinicians should prioritize adherence and postprandial glycemic control, as the latter critically mediates cognitive recovery.

The practical implications for older adults remain preliminary due to the scarcity of focused research. Emerging evidence suggests that older adults may respond differently to interruption frequencies compared to younger cohorts. Zheng et al. (2024) showed that a single 30-min bout of moderate-intensity walking was highly efficacious in interrupting SE. Maybe a single, longer bout of moderate-intensity activity yields greater cognitive benefits than frequent short breaks. Given the preliminary nature of these findings, we recommend that SE interruption for older adults be tailored to individual physical capacities and that practitioners prioritize safety and adherence over high-frequency protocols until more robust evidence is available.

In summary, while active SE interruption shows promise as a potent catalyst for acute brain health, there is no one-size-fits-all prescription. The effectiveness of these interventions is modulated by the type of activity, its intensity, and the characteristics of the target population. Furthermore, because most of the included studies focused on immediate post-intervention effects, these implications should not be interpreted as definitive clinical guidelines for chronic cognitive health. Future research is required to determine whether the cumulative effect of these acute bouts leads to sustained neuroprotective benefits.

### 4.4 Future research prospects

First, current evidence has mainly focused on acute effects, with few long-term randomized controlled trials demonstrating the persistent or cumulative benefits of these interruptions. Future research should include a longer intervention and follow-up period to examine the long-term cognitive effects of various interruption programs. The scarcity of evidence regarding chronic effects primarily stems from methodological barriers. In long-term interventions, maintaining high participant compliance with high-frequency interruption protocols in free-living environments is inherently challenging, often leading to intervention fatigue. Furthermore, accurately monitoring these specific micro-behaviors over extended periods requires continuous objective assessment, which significantly increases the resource burden and risk of data attrition compared to acute, laboratory-controlled studies. Second, the studies were conducted on healthy young people, and there is little data available for older adults, the clinical population, children and adolescents, and specific occupational groups. More research is desperately needed in these disparate populations and settings, especially for individuals who do very high amounts of mentally passive SE. Third, there is notable heterogeneity across studies in the types of interruption protocols used, the cognitive assessments used to assess the effects of interruption, and the participant characteristics included, thus precluding a quantitative synthesis (i.e., a meta-analysis) in this review. Future research could further explore this field by conducting numerous high-quality randomized controlled trials to identify the most effective ways to help different populations. This allows for more standardized intervention protocols and enables meaningful quantitative syntheses and comparisons.

### 4.5 Limitations of the article

Possible shortcomings of this article include the high heterogeneity across the included studies in interventions, participants, and outcomes, which makes a quantitative synthesis difficult, and an inability to explain the conclusions fully. Second, only a few original studies have compared different groups of SE interruption, so it is hard to know whether differences are related to how often, for how long, or what kinds of SE interruption they had. Furthermore, the small cumulative sample size limits the generalizability of our findings. While common in resource-intensive laboratory research and supported by the high internal validity of crossover designs, these small, homogeneous cohorts render the evidence preliminary. Future large-scale, multi-center randomized controlled trials with diverse populations are needed to validate the scalability of these cognitive benefits.

## 5 Conclusion

In summary, PA breaking up SE acutely improves cognitive function and promotes brain health, but current evidence is insufficient to determine its long-term effects on cognition. Therefore, high-quality research is urgently needed to explore further its potential for sustained cognitive health improvement through extended longitudinal interventions and follow-up periods.

## Data Availability

The original contributions presented in the study are included in the article/supplementary material, further inquiries can be directed to the corresponding author.
